# Release of CGRP from mouse brainstem slices indicates central inhibitory effect of triptans and kynurenate

**DOI:** 10.1186/1129-2377-15-7

**Published:** 2014-02-08

**Authors:** Charlotte Kageneck, Barbara E Nixdorf-Bergweiler, Karl Messlinger, Michael JM Fischer

**Affiliations:** 1 Institute of Physiology and Pathophysiology, University of Erlangen-Nuremberg, Universitaetsstrasse, 91054 Erlangen, Germany

**Keywords:** Headache, Migraine, Neuropeptide, Nociception, 5-HT receptor

## Abstract

**Background:**

CGRP is contained in a substantial proportion of unmyelinated trigeminal neurons innervating intracranial tissues. Previously, we have described a hemisected rodent scull preparation and later the intact trigeminal ganglion to measure stimulated CGRP release from trigeminal afferents.

**Methods:**

Here, we establish a preparation for examining CGRP release from central trigeminal terminals using single fresh slices of the mouse medullary brainstem.

**Results:**

Basal and stimulated amount of CGRP substantially exceeded the detection level. Experiments were designed as matched pairs of at least six brainstem slices per animal. Stimulation with high potassium induced calcium-dependent and reversible CGRP release. Capsaicin stimulation of TRPV1 provoked concentration-dependent CGRP release. The anti-migraine drug naratriptan did not inhibit capsaicin-induced CGRP release from peripheral terminals but inhibited the release from brainstem slices. The glutamate antagonist kynurenate showed a similar pattern of site-specific inhibition of CGRP release.

**Conclusions:**

As observed earlier for other drugs used in the treatment of migraine this indicates that the central terminals in the spinal trigeminal nucleus may be the main site of action. The preparation allows evaluating the trigeminal brainstem as a pharmacological site of action.

## Background

The neuropeptide calcitonin gene-related peptide (CGRP), a potent vasodilator in all mammals including humans, is found in a substantial percentage of trigeminal afferents. CGRP is released upon activation of peptidergic afferent neurons in animals [1] and also in humans, demonstrated by electrocoagulation of the trigeminal ganglion, where flushing of patients was correlated with elevated CGRP plasma levels [2]. Also trigeminal inflammation inside the blood brain barrier is sufficient to elevate venous CGRP outflow [3], but there is discussion regarding the functional significance of resting as well as elevated blood CGRP levels. Quantifying mass activation of primary sensory neurons via the release of expressed neuropeptides is an established experimental technique [4]. The neuropeptides are transported to all parts of the sensory neurons and are thus found not only in the cell body but also in the peripheral and central axons. Accordingly, CGRP release can be stimulated from these locations as previously shown throughout the body for peripheral projections of afferents [5], DRG neurons [6] and their central projections [7]. Various tissues innervated by trigeminal afferents, e.g. the tooth pulp, have been probed using CGRP release measurements [8]. For investigating the headache-relevant trigeminal system, we have previously established such a preparation for terminals in the dura mater of the hemisected rodent scull [9], and for the freshly dissected intact rodent trigeminal ganglion [10]. The mentioned preparations have been used to investigate functional aspects of the trigeminal system [11,12]. Compared to previous attempts to measure CGRP from the trigeminal brainstem, which have used at least one animal for a single data point, we present CGRP release from a single mouse trigeminal brainstem slice. The first synaptic relay site in the spinal trigeminal nucleus of the medullary brainstem is of particular interest, as it seems to be the key for the preferential action of CGRP and CGRP receptor antagonists [12]–[16]. Together with the CGRP release preparations described previously, this allows to investigate the effect of chemical stimuli and antagonists on trigeminal afferents at all possible sites of action. We employed this preparation to examine if there are site-specific mechanisms of action.

## Methods

All procedures were performed according to the German guidelines and regulations of animal care and welfare and approved by the responsible Animal Care Authority of the local district government (Ansbach, Germany). Experiments were carried out in accordance with the European Communities Council Directive of 24 November 1986 (86/609/EEC). For brainstem slices, home bred C57BL/6 mice of both sexes, aged 9–25 days, were used due to sufficient preparation size and experience regarding the vitality of slices for electrophysiological experiments. Mice were decapitated during inhalation anesthesia with halothane or sevofluorane. The medullary brainstem was dissected and cut in ice-cold artificial cerebrospinal fluid (ACSF, in mM: 87 NaCl, 2.5 KCl, 0.5 CaCl_2_, 7 MgCl_2_, 1.25 NaH_2_PO_4_, 25 NaHCO_3_, 75 sucrose and 30 D-glucose, pH 7.4, saturated with 95% O_2_ and 5% CO_2_). After cutting serial transverse slices on a vibrating blade microtome (VT1000S or VT1200S, Leica Biosystems Nussloch GmbH), the sections were transferred into synthetic interstitial fluid (SIF, in mM: 107.8 NaCl, 26.2 NaCO_3_, 9.64 Na-gluconate, 7.6 sucrose, 5.55 glucose, 3.48 KCl, 1.67 NaH_2_PO_4_, 1.53 CaCl_2_ and 0.69 MgSO_4_[17]. No difference was observed when slicing was performed in SIF instead of ACSF. Slices were cut within a range of 3 mm, extending from cervical C2 segments to the rostral medullary brainstem (obex). Slices of 150 μm appeared too fragile for the further experimental handling. Slices finally evaluated had a thickness within the range of 200–400 μm; we recommend the upper half of this range. All slices were captured and transferred on a micromesh into a 96 well plate filled with SIF. The plate was placed in a 37°C bath for 30 min before the experimental stimulation. Mouse trigeminal ganglia and mouse hemisected sculls were prepared as previously described [9,10].

Every brainstem slice was placed in a separate well and held at 37°C. An incubation step consisted of adding 125 μl carbogen-saturated SIF for 5 min, at the end of this period acquisition of 100 μl to determine CGRP content, removal of the remaining fluid, and a single wash with 125 μl before adding fluid for the next incubation period. In the first two steps basal CGRP release was determined, in the third step the preparations were stimulated, the fourth step was used to evaluate recovery. Adjacent slices were used for the different experimental groups to allow matched-pairs comparison. This matched-pairs comparison reduces the statistical effect of inter-day variation of the EIA. A maximum of eight slices per animal was evaluated. The increase in CGRP release evoked in pairs of identically stimulated adjacent slices was correlated (Capsaicin 100 nM, R = 0.64, p = 0.004, n = 19 pairs from 11 animals). Therefore, the provided n indicates the number of slices. However, all presented experimental groups contain at least four independent animals and days. The CGRP content of the samples was determined using a commercial sandwich enzyme immune assay kit for human CGRP, which is crossreactive to mouse CGRP (SPIbio, France). This technique was previously published in detail [18]. Briefly, the samples are incubated with precoated capture and acetylcholin-esterase-conjugated tracer antibody overnight, washed and developed with Ellman's reagent, quantified at 405 nm. The assay has a  Limit of detection  of 5 pg/ml for CGRP, all values measured in this study were substantially above this level. CGRP content of brainstem slices was measured as described previously [10]. Briefly, preparations were sliced in calcium-free SIF and homogenized using an Ultra-Turrax T8 (IKA Works, Staufen, Germany), preceded and followed by a 10 min period in 1 ml of 2 M acetic acid at 95°C. The suspension was centrifuged for 30 min at 10,000 g. The CGRP content of the supernatant was measured after titration to neutral pH. Blank and CGRP-spiked controls showed that the titrated acetic acid and the resulting 1330 mosmol for overnight antibody incubation had no effect on the CGRP measurements. In separate experiments with an identical experimental protocol, 100 μl of elution fluid was used to determine LDH activity with the LDH Cytotoxicity Assay Kit (Cayman, Ann Arbor, MI, USA) as described by the manufacturer.

The extracellular solution with KCl 40 mM was generated by isoosmolar substitution of sodium chloride by potassium chloride. Capsaicin and Kynurenate was obtained from Sigma-Aldrich (St. Louis, MO, USA), N-(4-t-Butylphenyl)-4-(3-chloropyridin-2-yl)tetrahydropyrazine-1(2H)-carboxamide (BCTC) from Biotrend (Cologne, Germany). Stock solutions were prepared in ethanol and freshly diluted for the experiment, the resulting final ethanol concentration was ≤ 0.01%. Naratriptan was obtained from Glaxo Wellcome (London, United Kingdom) and freshly diluted in SIF.

Statistical analysis was performed with STATISTICA (StatSoft, Tulsa, OK). Subsequent measurements within the same slices were tested with the Wilcoxon matched pairs test. The stimulation-induced changes of CGRP release between adjacent slices of different experimental groups were also compared with the Wilcoxon matched pairs test. Two-way analysis of variance was used to evaluate two or more groups at two or more time points. A concentration-response profile was fitted a Boltzmann function with a baseline fixed to zero in Origin (OriginLab, Massachusetts). Panels were compiled in Origin and arranged in CorelDraw (Corel, Ontario). Data are presented as mean ± SEM, p < 0.05 was considered significant.

## Results

**Figure 1:**
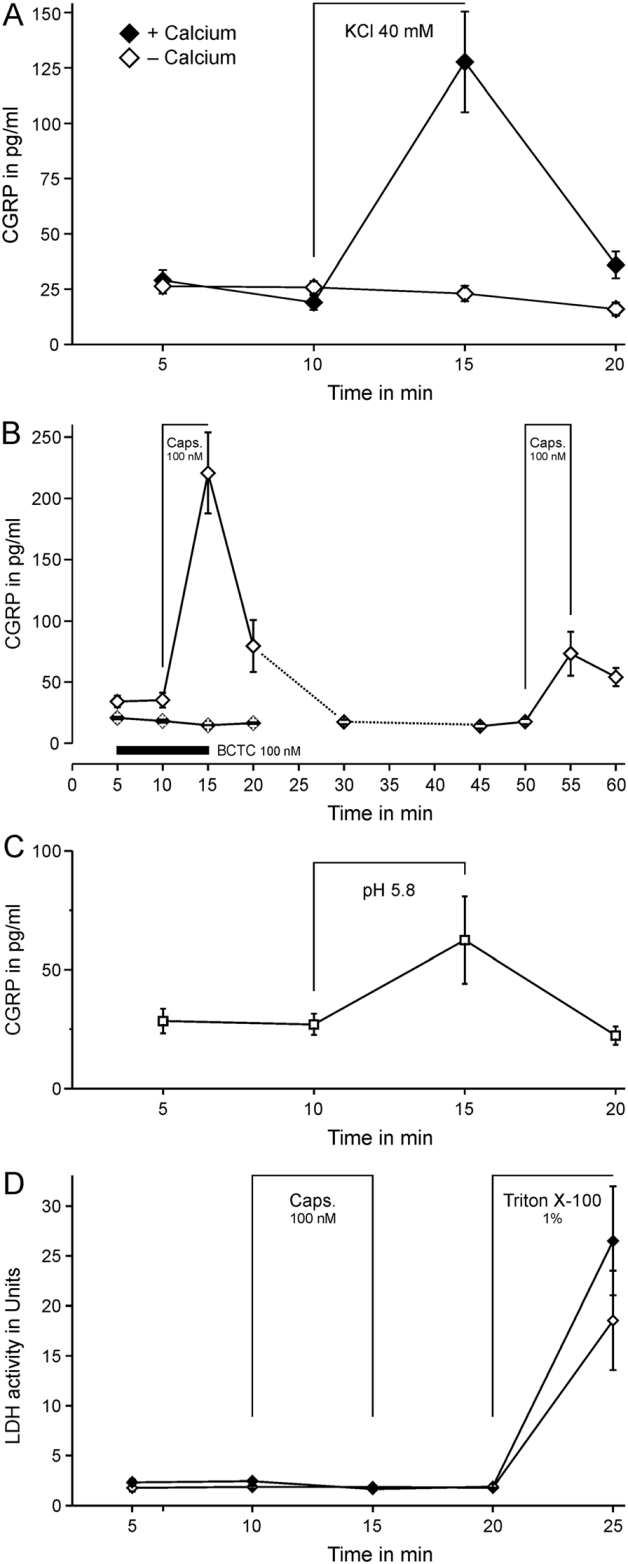
**Stimulated calcitonin gene-related peptide (CGRP) release from single mouse brainstem slices. A)** Potassium-induced depolarisation for 5 min induced CGRP release in a reversible manner (n = 11). In adjacent slices, no CGRP release was observed by the same stimulation in the absence of calcium. **B)** Capsaicin-induced stimulation of CGRP release. The second stimulation induced less CGRP release (n = 11). In the presence of the TRPV1 antagonist BCTC 100 nM (duration indicated by black bar, closed symbols, n = 8) capsaicin-induced release was abolished. **C)** Acidic extracellular solution of pH 5.8 stimulated CGRP release from brainstem slices (n = 11). **D)** LDH-activity assay from brainstem slices, treated as described above. In the eluate, a low and constant LDH activity was observed throughout, treatment with capsaicin did not alter LDH activity. Triton-X 100 employed as a positive control increased LDH activity in all samples (*); there was no difference between the brainstem slices previously treated with capsaicin 100 nM (filled symbols) and those without (open symbols, n = 4 each). No increase in LDH activity was observed 1, 2, 4 and 8 h after preparation (n = 3, not shown). Data are mean ± SEM.

Basal CGRP release from mouse brainstem slices within 5 min was 25 ± 1 pg/ml (n = 133, slices of 300–400 μm, pooled from the first 5 min). CGRP release was not correlated to the thickness in the tested range of 200–400 μm (R = 0.01, product-momentum correlation). Compared to baseline, depolarization by potassium chloride 40 mM increased CGRP release by 109 ± 23 pg/ml (p = 0.003, n = 11, Wilcoxon). This recovered by 84% within the subsequent 5 min. No potassium-stimulated CGRP release was observed in the absence of extracellular calcium (p = 0.21, n = 10, Wilcoxon, + EGTA 5 mM, Figure 1A). Capsaicin 100 nM increased CGRP release from 35 ± 6 pg/ml to 221 ± 33 pg/ml (p = 0.003, n = 11, Wilcoxon). The quick recovery led us to investigate repetitive stimulation of the same sample. A second stimulation with capsaicin 100 nM 40 min later increased CGRP by 56 ± 18 pg/ml (p = 0.008), which is 30% of the first stimulation (Figure 1B). Addition of the TRPV1 antagonist BCTC 100 nM after 5 min had no effect on its own but abolished the capsaicin-evoked response compared to experiments without BCTC (p = 0.33, n = 8, Wilcoxon). Acidic SIF (pH 5.8) also increased CGRP release by 26 pg/ml (p = 0.004, n = 11, Wilcoxon, Figure 1C), but less compared to capsaicin, which was therefore preferred for further experiments. The vitality of the tissue was probed by quantifying extracellular LDH activity. The samples were subjected to four consecutive 5-min elution steps in SIF, a constant LDH activity was observed throughout (n = 8). In four of these samples capsaicin 100 nM was added in the third period, which did not alter LDH activity. As positive control, Triton X-100 1% added to the fifth period increased LDH activity substantially (ANOVA, F_(1,6)_ = 24.6, p = 0.003, Figure 1D). No difference was observed in samples previously exposed to capsaicin compared to those treated without capsaicin (p = 0.60, HSD-post-hoc test). We thus measured LDH activity at periods of 1, 2, 4 and 8 h after preparation and observed no increase (15 min of sampling in SIF, n = 3, data not shown).

**Figure 2:**
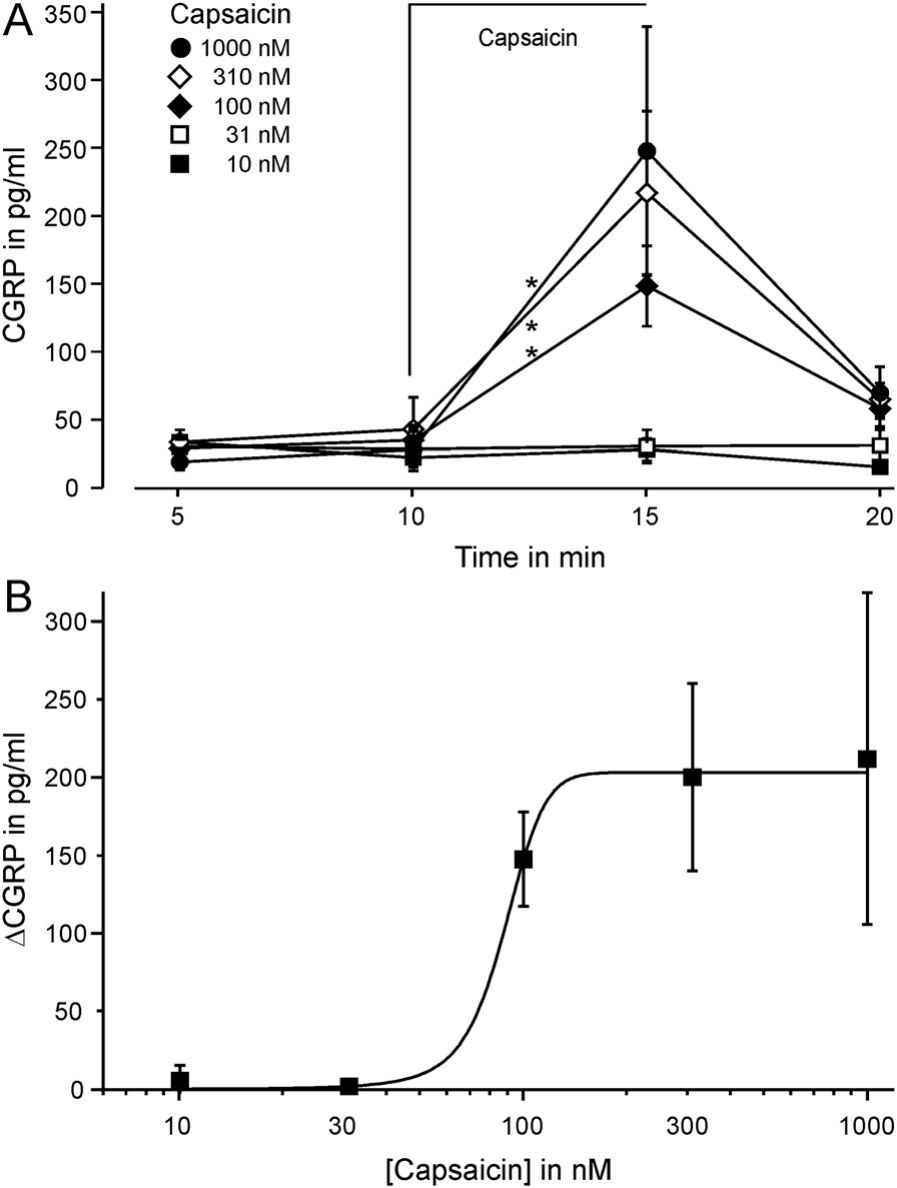
**Concentration-dependence of capsaicin-induced CGRP release. A)** Time course of the experiments, showing the concentration-dependent increase in CGRP for capsaicin ≥ 100 nM, which largely recovered within the subsequent 5 min (n = 6–14). **B)** For the increase in CGRP release (ΔCGRP), calculated between the values at 10 and 15 min, a fitted dose–response function had an EC_50_ of 88 ± 5 nM. Data are mean ± SEM. *p < 0.05.

Capsaicin stimulation was tested in the range of 10–1000 nM. A significant increase in CGRP release was observed for capsaicin doses of 100 nM and above (p = 0.009, p = 0.028 and p = 0.012, n = 6–15, Wilcoxon). Concentration-dependent CGRP release by capsaicin had an EC_50_ of 88 ± 5 nM (Figure 2). A 400 μm thick mouse brainstem slice had a wet weight of 3.7 ± 0.5 mg and a CGRP content of 1217 ± 180 ng/g (n = 5). Thus, capsaicin 1 μM released about 0.6% of the total CGRP content within five minutes.

**Figure 3:**
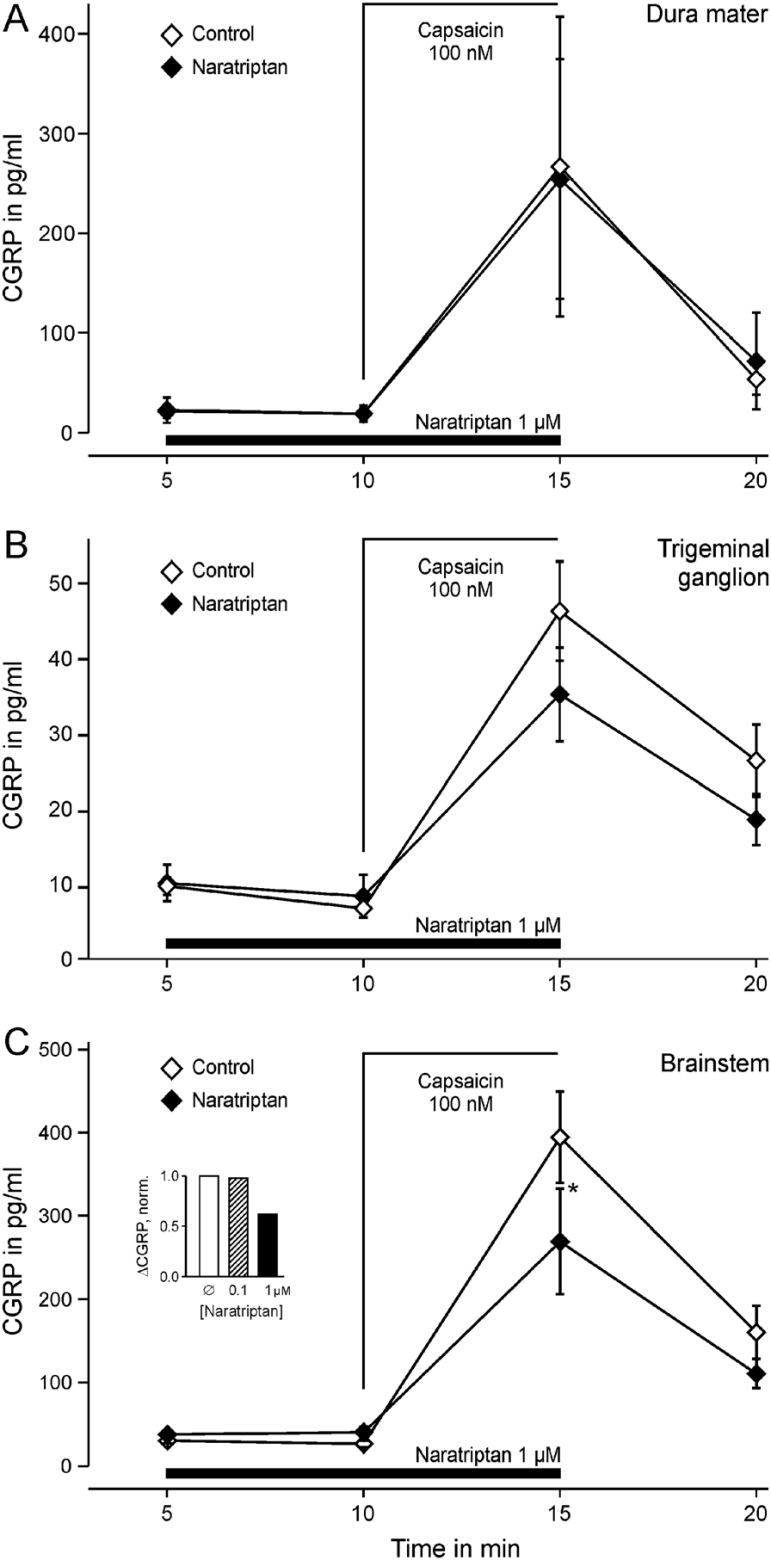
**Naratriptan action at different parts of mouse trigeminal afferent neurons. A)** Capsaicin-stimulated CGRP release from the dura mater was not altered by naratriptan (hemisected scull preparation, n = 12 each). **B)** Capsaicin-stimulated CGRP release from isolated intact trigeminal ganglia. Naratriptan did not induce a significant difference (n = 12 each). **C)** Capsaicin-stimulated CGRP release from brainstem slices was significantly reduced in the presence of naratriptan (n = 13), applied during and before capsaicin application as indicated. Naratriptan alone did not alter basal CGRP release from the dura, the ganglion or the brainstem. The inset shows the inhibition of the increase in CGRP release by naratriptan. Data are mean ± SEM. *p < 0.05.

**Figure 4:**
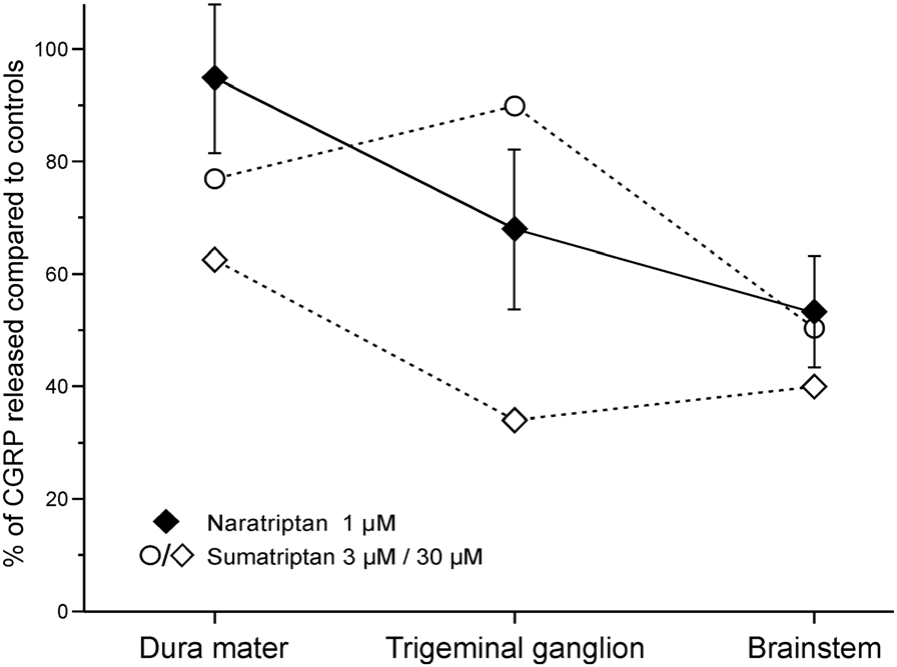
**Triptans inhibit CGRP release in a location-dependent manner.** Open symbols show the inhibition of potassium-stimulated CGRP release by sumatriptan (data taken from [11]). Closed symbols represent the inhibition of capsaicin-induced CGRP release by naratriptan observed in the current study. There is a correlation between the site of application and percent of inhibition by naratriptan (R = -0.48, p = 0.004, Spearman correlation). Data are mean ± SEM.

The effect of naratriptan on CGRP release from the peripheral projections, cell bodies and central projections of trigeminal neurons was tested employing a preparation of the dura in the hemisected skull, the isolated intact trigeminal ganglion and brainstem slices from mice. Capsaicin induced CGRP release of 248 ± 42 pg/ml from the hemisected scull, 33 ± 6 pg/ml from the isolated intact trigeminal ganglion and 363 ± 80 pg/ml from brainstem slices (p = 0.002, p = 0.002 and p = 0.003, n = 12–13, Wilcoxon, Figure 3). Application of naratriptan 1 μM alone did not alter basal CGRP release compared to controls in all three preparations. In the hemisected skull the presence of naratriptan 1 μM did not alter capsaicin-evoked CGRP release (p = 0.43, n = 12, Wilcoxon). In the trigeminal ganglion, evoked CGRP release in the presence of naratriptan was 68% of the control (p = 0.09, n = 12, Wilcoxon) and in the brainstem slices naratriptan reduced the evoked CGRP release to 55% (p = 0.047, n = 11, Wilcoxon). In the presence of naratriptan 0.1 μM CGRP release was 98% of controls (p = 0.50, n = 10, Wilcoxon). Naratriptan 10 μM released 107% compared to control experiments (n = 5). The pattern of the remaining CGRP release in the presence of naratriptan 1 μM of the present study is overlayed with previous results in Figure 4[11].

**Figure 5:**
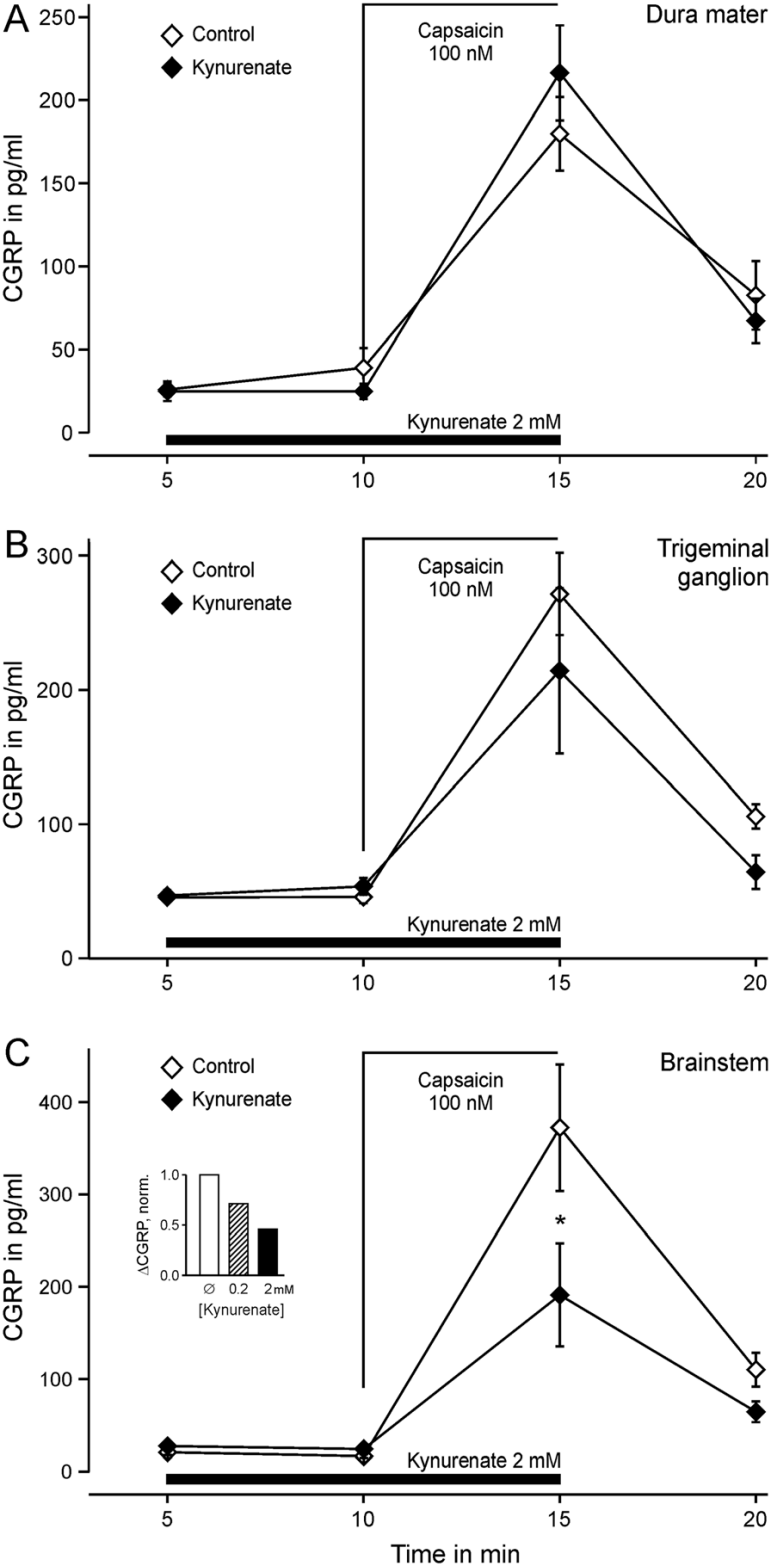
**Kynurenate action at different parts of mouse trigeminal afferent neurons. A)** Capsaicin-stimulated CGRP release from the dura mater was not altered by kynurenate (hemisected scull preparation, n = 8 each). **B)** Capsaicin-stimulated CGRP release from isolated intact trigeminal ganglia. Kynurenate did not induce a significant difference (n = 8). **C)** Capsaicin-stimulated CGRP release from brainstem slices was significantly reduced in the presence of kynurenate 2 mM (n = 15), applied during and before capsaicin application as indicated. Kynurenate alone did not alter basal CGRP release from the dura, the ganglion or the brainstem. Concentration-dependent inhibition of the increase in CGRP release by kynurenate is shown in the inset. Data are mean ± SEM. *p < 0.05.

In a similar fashion, the effect of the glutamate receptor antagonist kynurenate on CGRP release was probed at different sites within the mouse trigeminal system. Kynurenate was applied in the second step and did not alter basal CGRP release in all three preparations. In the hemisected skull preparation, capsaicin-stimulated CGRP release from the dura mater was not inhibited by kynurenate 2 mM (136% of controls, Figure 5). Capsaicin-stimulated CGRP release from isolated intact trigeminal ganglia in the presence of kynurenate 2 mM was 70% of the controls (p = 0.07, n = 8, Wilcoxon). However, capsaicin-stimulated CGRP release from brainstem slices was significantly reduced by kynurenate 2 mM to 46% of the controls (p = 0.036, n = 15, Wilcoxon). In the presence of kynurenate 0.2 mM, CGRP release was 71% of the controls (p = 0.09, n = 11, Wilcoxon), kynurenate 20 mM released 135% of the controls (n = 4).

## Discussion

We have established a preparation in which we assessed stimulation-induced, reversible and robust CGRP release from single mouse medullary brainstem slices. Naratriptan and kynurenate inhibited capsaicin-induced CGRP release from the medullary brainstem but not from peripheral terminals innervating the dura mater.

Shortly after CGRP was discovered [19], its presence in primary sensory afferents [20], and in particular trigeminal neurons was described [21]. Compared with other neuropeptides, CGRP is the most widely expressed neuropeptide in sensory afferents [22]. CGRP is produced by a large proportion of small- and intermediate-sized trigeminal afferents and transported from the somata to the peripheral and the central terminals. In the spinal trigeminal nucleus (medullary dorsal horn), which is the central projection site of most of nociceptive trigeminal afferents, CGRP immunoreactivity is found preferably in the superficial layers I-II [23]. Dorsal rhizotomy or neonatal capsaicin treatment depleted spinal CGRP content, which indicates the exclusive presence in primary afferents [24,25]. This is important for the presented preparation, in which afferent fibers comprise a small minority of the tissue. Despite the large number of cell types contained in the slices, specificity is not only gained by sampling CGRP-containing neurons, but also by stimulation with subpopulation-specific agonists like capsaicin. Stimulation with acidic pH, which typically involves TRPV1 [26], led only to a limited release of CGRP compared to capsaicin.

### Comparison to previously published preparations

All previous studies investigating CGRP release have used brainstem tissue of at least one animal [11,12,27], some even multiple animals [28]. In contrast, the presented preparation uses less brainstem tissue with a smaller volume to measure a robust signal. This allowed performing multiple experiments from slices of a single animal, including a matched pairs design with adjacent slices. In contrast, compared to CGRP release from lumbar spinal cord cut by a tissue chopper into small pieces [29], the method presented here is less disruptive. A limitation of the method is the restriction to CGRP-expressing neurons; no similar index substance is available for non-peptidergic neurons. In conclusion, several advantages qualify this preparation to screen effects on the activation of peptidergic central trigeminal terminals. Certainly, the brainstem slices show undiminished vitality throughout the experiments, which is confirmed by the employed LDH assay. Brainstem slices of similar thickness prepared with a similar protocol for electrophysiological recordings remain usable for several hours.

Capsaicin has been shown to stimulate CGRP release from rat spinal cord slices using a radioimmunoassay [7,30]. Immunohistochemistry has been used to show the release of neuropeptides by depletion [31], but this procedure requires excessive trigeminal ganglion stimulation to generate any change in the trigeminal nucleus. This is not surprising as there might be reuptake, and in our hands a strong stimulation for five minutes only releases a small fraction of the neuropeptide content. Microprobes have been used to detect the release of neuropeptides such as substance P [32,33]. This semi-quantitative method with high sensitivity and spatial resolution has occasionally been employed for CGRP release measurements but is limited by reliable fixation of CGRP antibodies to the glass probes [34].

### Practical considerations

We initially varied slice thickness to investigate the amount of required tissue. Within the investigated range of 200–400 μm, no association of basal or stimulated CGRP release to slice thickness was observed. As a result, we considered it acceptable to pool the data from different slice thicknesses. Thinner slices (150 μm) were fragile and difficult to handle imposing a lower limit. Tissue poses some diffusion barrier as a function of molecular weight and size; in our preparation the 37 amino acid large CGRP molecule can be expected to diffuse slower than the solutions containing the pharmacological substances. The distance of released CGRP to the cutting surface is less than to the dorsal surface of the brainstem, so we assume that direct diffusion of CGRP from the cut surface has the largest contribution to the total release. This is in line with the observation that released CGRP does not vary with slice thickness. Various duration of the single steps have been used in the literature. We have previously observed a positive association between duration and CGRP content, indicating an ongoing release. Based on the signal, a range of 2–10 min seems practical, a period of 5 min was chosen for the present study as this was the most common sampling time used previously. The presented release from brainstem slices allows choosing the region of interest. Our variation of slice thickness and the lack of precise histology-based allocation for every used slice did not allow generating a histogram of CGRP release reflecting the position in cranio-caudal direction. It may be assumed that there is an association with the density of CGRP, which has been investigated down to the C1 level with much thinner slices before [35]. In the case of capsaicin stimulation this also relies on the assumption that the overlap between the capsaicin receptor TRPV1 and CGRP does not vary with the position.

Activation of neurons by a nociceptive-specific agonist like capsaicin activates a smaller population of neurons compared to the generally depolarizing potassium chloride. Nevertheless the amount of CGRP released after capsaicin was higher than after KCl, which argues for a higher or prolonged rise of intracellular free calcium upon activation of TRPV1 receptor channels. The concentration of potassium 40 mM was chosen based on results from other preparations, sufficient depolarization for admittance of calcium occurs around potassium 15 mM, a plateau of activation is typically observed at potassium 80 mM. In case the investigation of knockout mice is not an issue, the higher expression of CGRP in rats might favor these for the presented approach.

### Differential triptan effects

Different triptans and selective agonists for the 5-HT1 receptor subtypes have been investigated in the trigeminal system [11]. Aggregating the results of both applied sumatriptan concentrations used in their study reveals a similar pattern as we observed for naratriptan; the inhibition at the central site is more prominent compared to the peripheral site. This might have been obfuscated by the variation at the trigeminal ganglion, as this has the smallest signal of the three preparations. Taking the average of both sumatriptan doses fits our results with naratriptan well (Figure 4). Naratriptan has an EC_50_ below 100 nM on both 5HT_1B_ and 5-HT_1D_ receptors [36]. 5-HT_1B_ and 5-HT_1D_ receptors are present in more than 80% of human trigeminal ganglion neurons and the majority of CGRP-containing cells are positive for both receptors [37]; similar expression and colocalization was observed in rats [38]. The molecular mechanism of the triptan action downstream of 5-HT receptors is not fully understood, but at least it seems to be comprised of several independent effects including the inhibition of voltage-gated sodium channels, suppression of voltage-gated calcium currents and a hyperpolarizing shift in the voltage-dependence of potassium currents [39].

### Differential kynurenate effects

Effects of the endogenously occurring kynurenate are biochemically linked to 5-HT, as both are synthesized from tryptophan, and both pathways interact [40]–[43]. Kynurenate is an antagonist of NMDA, AMPA and kainate receptors [44], and therefore frequently used in experimental models to investigate ionotropic glutamate receptors effects. The concentration of 2 mM kynurenate is frequently used; a sensitization of capsaicin-induced release observed at 20 mM occurs probably through a different target. Glutamate receptor inhibition has been suggested as potential therapeutic mechanism for headaches. Activity induced by electrical stimulation of the trigeminal ganglion, resulting in increased c-fos immunoreactivity in the caudal trigeminal nucleus, was almost abolished by kynurenate infusion in rats [45]. Kynurenate was shown to inhibit the delayed trigeminal activation induced by glyceroltrinitrate, using CamKII or cFos as a marker [46,47]. Combined inhibition of AMPA and kainate receptors was effective as treatment for migraine [48]. All ionotropic glutamate receptors were found in the superficial laminae of the trigeminal nucleus [49]. Subunits of the NMDA receptor were found on about 20% of rat trigeminal sensory neurons [50,51], also AMPA, kainate and metabotropic glutamate mGluR5 receptors were found in the rat trigeminal ganglion [52,53]. The somata of rat trigeminal sensory neurons were shown to respond to stimulation with AMPA, NMDA, kainate and mGluR agonists [54]. For NMDA receptors, sensitization of TRPV1 via phosphorylation was evidenced as potential downstream mechanism in trigeminal sensory neurons [55]. Since the central terminals of primary afferents are the most important if not the only source for CGRP in the medulla, it is likely that presynaptic glutamate receptors are responsible for the glutamate effect. In the presented experiments, capsaicin-released glutamate might contribute to the calcium-dependent CGRP release via presynaptic glutamatergic autoreceptors [56]. The activation of calcium-permeable NMDA receptors could directly admit calcium to central afferent terminals, providing a secondary fraction of calcium in addition to the calcium entering via TRPV1 upon capsaicin stimulation. At the central terminals this contribution seems significant, given that kynurenate blocked about half of the capsaicin-stimulated release in brainstem slices. It should be noted that kynurenate could exert its effects through other targets than glutamate receptors, e.g. the G-protein-coupled receptor GPR35 or alpha7 nicotinic acetylcholine [57]–[60]. The production of glutamate receptors in the trigeminal ganglion suggests a transport of glutamate receptors peripherally and centrally. Since kynurenate did not inhibit CGRP release in the dura mater, it might by hypothesized that the required cascades are only fully assembled in presynaptic terminals, but not complete at other parts of the trigeminal afferents.

Administration of CGRP has facilitated glutamatergic transmission [16]. The basis for this cooperative effect of CGRP and glutamate is not clear. We speculate that CGRP itself might facilitate glutamate release, which would fit to the immunohistochemical observation that CGRP receptor components are localized presynaptically on primary afferent terminals, but not on second order neuron cell bodies in the Sp5C [61].

## Conclusions

This article presents a CGRP release preparation from mouse brainstem slices and therefore extends the release preparations reported by the group previously. A triptan and a glutamate receptor antagonist both inhibited the CGRP release from the brainstem, whereas there was only a tendency towards inhibition in the trigeminal ganglion and no effect in the dura mater. This shows that the three preparations allow investigating the site of action throughout the first neuron of the trigeminal system.

## Abbreviations

ACSF: Artificial cerebrospinal fluid; CGRP: Calcitonin gene-related peptide; SIF: Synthetic interstitial fluid; TRPV1: Transient receptor potential, vanilloid type 1.

## Competing interests

The study received support by the Emerging Fields Initiative of the Friedrich-Alexander-University. The authors declare no conflict of interest.

## Authors’ contributions

CK performed the experiments and wrote the manuscript; the present work was performed to obtain the degree  Dr. med.’ at the  Friedrich-Alexander Universität Erlangen-Nürnberg (FAU)’. BS-B sliced brainstems, KM wrote the manuscript and supervised the project. MJMF designed the study, analyzed the data and wrote the manuscript. All authors read and approved the final manuscript.
